# Blind Tone-Mapped Image Quality Assessment Based on Regional Sparse Response and Aesthetics

**DOI:** 10.3390/e22080850

**Published:** 2020-07-31

**Authors:** Zhouyan He, Mei Yu, Fen Chen, Zongju Peng, Haiyong Xu, Yang Song

**Affiliations:** Faculty of Information Science and Engineering, Ningbo University, Ningbo 315211, China; hezhouyan@nbu.edu.cn (Z.H.); chenfen@nbu.edu.cn (F.C.); pengzongju@nbu.edu.cn (Z.P.); xuhaiyong@nbu.edu.cn (H.X.); songyang@nbu.edu.cn (Y.S.)

**Keywords:** high dynamic range image, tone-mapping, image quality assessment, regional sparse response, aesthetics, entropy weighting

## Abstract

High dynamic range (HDR) images give a strong disposition to capture all parts of natural scene information due to their wider brightness range than traditional low dynamic range (LDR) images. However, to visualize HDR images on common LDR displays, tone mapping operations (TMOs) are extra required, which inevitably lead to visual quality degradation, especially in the bright and dark regions. To evaluate the performance of different TMOs accurately, this paper proposes a blind tone-mapped image quality assessment method based on regional sparse response and aesthetics (RSRA-BTMI) by considering the influences of detail information and color on the human visual system. Specifically, for the detail loss in a tone-mapped image (TMI), multi-dictionaries are first designed for different brightness regions and whole TMI. Then regional sparse atoms aggregated by local entropy and global reconstruction residuals are presented to characterize the regional and global detail distortion in TMI, respectively. Besides, a few efficient aesthetic features are extracted to measure the color unnaturalness of TMI. Finally, all extracted features are linked with relevant subjective scores to conduct quality regression via random forest. Experimental results on the ESPL-LIVE HDR database demonstrate that the proposed RSRA-BTMI method is superior to the existing state-of-the-art blind TMI quality assessment methods.

## 1. Introduction

High dynamic range (HDR) imaging, as a popular image enhancement technology, aims at recovering the detail information in bright and dark regions of images by fusing multiple low dynamic range (LDR) images with varying exposure levels [1]. Consequently, HDR images have a powerful ability to acquire almost all brightness ranges in natural scenes, and have attracted attention from various multimedia signal processing fields, such as HDR compression, streaming and display [2]. Moreover, due to the limitations on popularization of HDR display devices, tone-mapping operators (TMOs) have been successively developed to ensure the visualization of HDR images on traditional LDR displays, which reduce brightness dynamic range of images as much as possible without destroying the original structure of scenes [3]. Unfortunately, there are no completely suitable TMOs for converting HDR images, so that the relevant visual quality degradation phenomena (e.g., detail loss especially in the bright and dark regions and color unnaturalness) will be inevitably introduced into tone-mapped images (TMIs) [4]. To distinguish the generalization ability of different TMOs accurately, objective image quality assessment (IQA) of TMIs is one of the most challenging problems to optimize the HDR processing pipeline.

Up to now, a large number of perceptual IQA methods designed for LDR images have been proposed [5,6], and can be usually divided into three categories: full-reference (FR), reduced-reference (RR) and no-reference/blind (NR). The FR methods are guided by a distortion-free reference image. The RR methods only require a part of the reference image, while the NR/blind methods do not. Among the classical FR-IQA methods, the structural similarity method (SSIM) [5] is one of the most influential methods in academic communities, which measures the difference between the reference image and distorted image from brightness, contrast and structure. Evidently, these IQA methods for LDR images are not applicable to TMIs due to the specific truth that reference and distorted images have different dynamic range. To solve this problem, Yeganeh et al. [7] proposed the tone-mapped quality index (TMQI) by combining multi-scale structure fidelity and statistical naturalness in the grayscale domain. Although the TMQI method outperforms the existing IQA methods designed for LDR images in terms of predicting the quality of TMIs, there is still large room for further improvement, such as perceptual analysis in chrominance domain. Afterwards, a few improved versions based on the TMQI method were put forward. Ma et al. [8] revised the related feature components in the TMQI method for the accuracy of evaluation. Nasrinpour et al. [9] integrated the importance of salient regions into the TMQI method to further improve the evaluation performance. Besides, Nafchi et al. [10] expended the existing feature similarity (FSIM) [11] method to form the feature similarity index for tone-mapped images (FSITM). Song et al. [12] utilized the exposure condition segmentation and extracted perceptual features to predict the quality of TMIs. Unfortunately, considering that the reference HDR images are usually unavailable and unintelligible in many practical cases, the above FR-IQA methods designed for TMIs are prone to defeat despite the advanced performance on the benchmark TMIs database.

Obviously, the development of blind IQA (BIQA) methods is more challenging compared with FR-IQA methods due to the lack of a reference image. Generally, most BIQA methods designed for ordinary 2D images (2D-BIQA) are based on the framework of supervised learning, that is, several quality-aware features are extracted from images and quality regression is conducted via the model trained by machine learning or deep learning algorithms [13,14,15,16]. Among the diverse quality-aware features, natural scene statistics (NSS) based features play a significant role in evaluating 2D images degraded with single distortion or multiple distortions. Moorthy et al. [13] presented the distortion identification-based image verity and integrity evaluation (DIIVINE) method by exploring the statistics between the sub-band coefficients obtained from steerable pyramid decomposition. Zhang et al. [14] extracted the additional complex phase statistics on the basis of the DIIVINE method. Saad et al. [15] and Mittal et al. [16] developed the BLIINDS-II and BRISQUE methods by using the NSS of discrete cosine transform (DCT) coefficients and mean subtracted contrast normalized (MSCN) coefficients, respectively. Moreover, there exist some aesthetic IQA methods. For example, Sun et al. [17] proposed an alternative set of features for aesthetic estimation based on a visual complexity principle. They extracted the visual complexity properties from an input image in terms of their composition, shape, and distribution. Mavridaki et al. [18] introduced five feature vectors for describing the photo’s simplicity, colorfulness, sharpness, pattern and composition to perform the aesthetic quality evaluation. Although these 2D-BIQA methods and aesthetic IQA methods have shown their performance superiority in predicting the quality of 2D images and aesthetic-related images addressed by common distortion types, e.g., blockiness, blurriness, noise and aesthetic drop, there is a large gap in predicting the quality of TMIs dominated by detail loss especially in the bright and dark regions and color unnaturalness. The reasons for performance deviation can be summarized as the following two aspects. First, NSS based features are extracted from the entire image or sub-band, and can be usually regarded as global features, so the relevant local features (e.g., local structure and texture information) are ignored. Remarkably, the detail loss of TMIs caused by structural degradation is mainly reflected in bright and dark regions of images. Another problem is that the extracted NSS features are based on luminance component of image, missing the crucial role of color information on the human visual system (HVS). Therefore, it is necessary to explore the special perceptual characteristics of TMIs to improve the performance of IQA methods.

Actually, some BIQA methods specialized for TMIs (TM-BIQA) have been presented in the past three years [19,20,21,22,23,24,25,26]. Gu et al. [19] designed a blind tone-mapped quality index (BTMQI) by analyzing information fidelity, naturalness and structure. Considering that the brightest and darkest regions of TMIs are prone to detail loss, Jiang et al. [20] proposed a blind TM-IQA (BTMIQA) method by combining the detail features with naturalness and aesthetic features. Kundu et al. [21] utilized the NSS features from the spatial domain and HDR gradient domain to form the HIGRADE method. Yue et al. [22] extracted multiple quality-sensitive features including colorfulness, naturalness, and structure to construct a TM-BIQA method. Jiang et al. [23] proposed a blind quality evaluator of tone-mapped images (BLIQUE-TMI) by considering the impact of visual information, local structure and naturalness on HVS, where the former two kinds of features are extracted based on sparse representation, and the other ones are derived from color statistics. Zhao et al. [24] proposed a method that is mainly based on local phase congruency, some statistical characteristics on the edge maps and opponent color space to measure the image sharpness, halo effect and chromatic distortion, respectively. Chi et al. [25] designed a new blind TM IQA method with image segmentation and visual perception, a feature clustering scheme was proposed to quantify the importance of features. Fang et al. [26] extracted features from global statistics model to characterize the naturalness and local texture features to capture the quality degradation. However, these TM-BIQA methods still have the following limitations: (1) The color information is completely ignored in the BTMQI and HIGRADE methods, and the aesthetic quality of TMIs cannot be evaluated in the BLIQUE-TMI method. (2) For the BTMIQA method, the extracted local features are too simple to characterize the visual perception for different brightness regions (DB-regions) in TMIs, and the detail loss phenomenon in regions of normal exposure is also omitted.

Towards a more accurate evaluation for TMIs, a blind TMI quality assessment method based on regional sparse response and aesthetics is proposed in this paper, denoted as RSRA-BTMI. The basic consideration of RSRA-BTMI is that we attempt to dig some quality-aware features from imaging and viewing properties of TMIs, i.e., we focus on exploring the specific perceptual characteristics for DB-regions in TMIs, so that extracting both local and global features to portray the detail loss and color unnaturalness. In summary, the main contributions of this paper are described as follows.

(1) Inspired by the viewing properties in visual physiology, i.e., the quality of images is perceived by HVS from global to local regions, multi-dictionaries are specially designed for DB-regions of TMIs and entire TMIs via dictionary learning. Moreover, the self-built TMIs training dataset for dictionary learning in this study is available for the further research demand.
(2) Each region is sparsely represented to obtain the corresponding sparse atoms activity for describing the regional visual information of TMIs, which is closely related to visual activity in the receptive fields of simple cells. In addition, a regional feature fusion strategy based on entropy weighting is presented to aggregate the above local features.
(3) Motivated by the fact that HVS prefers an image with saturated and natural color, the relevant aesthetic features, e.g., contrast, color fidelity, color temperature and darkness, are extracted for global chrominance analysis. Besides, residual information of entire TMIs is fully utilized to simulate global perception of HVS, and the NSS based features extracted from residual images are combined with the aesthetic features to form the final global features.

The rest of the paper is organized as follows: The proposed RSRA-BTMI method is described in Section 2. The performance comparison results of RSRA-BTMI and other BIQA methods are presented in Section 3. Finally, the conclusion is given in Section 4.

## 2. The Proposed RSRA-BTMI Method

**Figure 1:**
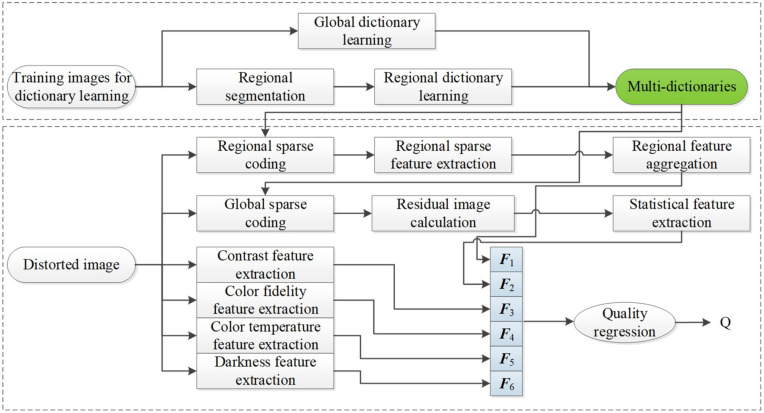
Framework of the proposed blind tone-mapped image (TMI) quality assessment method based on regional sparse response and aesthetics (RSRA-BTMI).

Figure 1 depicts the framework of the proposed RSRA-BTMI method, including regional sparse response feature extraction from DB-regions and global region of TMI in the sparse domain, and aesthetic features extraction for distinguishing color distortion. To be specific, to characterize the specific perceptual characteristics for DB-regions in TMIs, multi-dictionaries based on region segmentation via entropy are first learned to extract regional sparse response features, i.e., sparse atoms activity for each region and global reconstruction residual statistics. Moreover, aesthetic features including contrast, color fidelity, color temperature and darkness are extracted to portray color unnaturalness. All extracted features are formed into a feature vector to predict the quality of TMI through random forest (RF). The specific implementation of the RSRA-BTMI method is stated in the following subsections.

### 2.1. Multi-Dictionary Learning Based on Region Segmentation

Different TMOs will inevitably cause detail loss in DB-regions of TMIs and this kind of distortion usually affects the TMIs’ quality with specific means, which indicates the importance of detail information in the DB-regions to IQA of TMI, especially in the bright and dark regions [20]. In the proposed method, multi-dictionaries are first designed to obtain the regional sparse response features via regional sparse representation and global reconstruction residual calculation. Remarkably, this section is the foundation of the following feature extraction in the sparse domain. From the perspective of neurophysiology [27], when visual neurons receive the external stimuli, the information carried by the stimulus can be correctly perceived, while sparse representation is exactly consistent with the perceptual process of the visual signal. Moreover, according to the previous studies about visual signal processing, it has been proven that sparse representation can effectively match the visual perception characteristics of mammalian organism and describe the image signals with their sparsity and redundancy [28,29,30]. Therefore, sparse representation is used to identify the specific distortion of TMI in this study, i.e., regional and global detail loss.

#### 2.1.1. Constructing Dataset for Multi-Dictionary Learning

**Figure 2:**
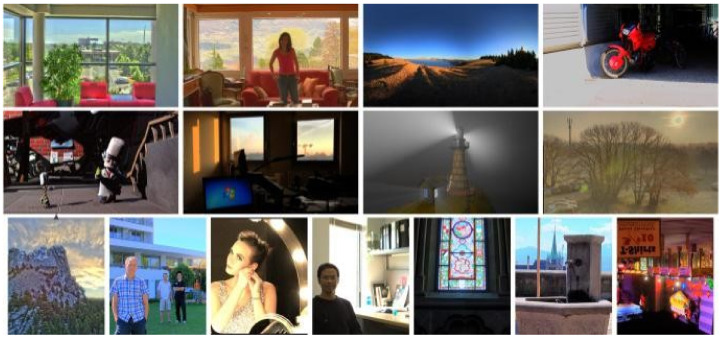
An example of the high dynamic range (HDR) image contents in the constructed TMI training dataset.

To obtain the perceptual features in the sparse domain, a novel TMI training dataset is constructed as the basic of multi-dictionary learning. Specifically, we selected 20 pristine HDR images with different kinds of scenes from existing HDR image datasets [31,32] and generated the corresponding distorted versions processed with 15 classical TMOs [33]. To avoid the same TMI datasets used in dictionary learning and quality assessment stages, the image contents contained in TMI training dataset were distinct from those in the subsequent-used benchmark database for objective quality assessment (i.e., ESPL-LIVE HDR database [34]), and Figure 2 depicts the partial scene contents, which includes the indoor, outdoor and night scenes. Moreover, we artificially eliminated some low-quality distorted image under extreme conditions, such as abnormal exposure, annoying noise and indelible artifacts, so as to construct the final TMI training dataset for multi-dictionary learning.

#### 2.1.2. TMI Segmentation for Multi-Dictionaries

**Figure 3:**
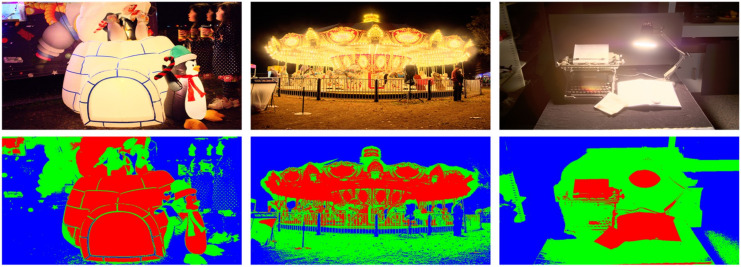
Three TMIs and their corresponding brightness segmentation images, where the parts of red, green and blue of images in the second row are the bright region, normal exposure region and dark region, respectively.

Inspired by viewing properties in visual physiology, HVS tends to perceive the detail information of TMIs from DB-regions, especially the bright and dark regions in an image. Therefore, an advanced brightness segmentation algorithm via entropy [35] is first applied to divide a TMI into three types of brightness regions, i.e., bright region, normal exposure region and dark region, denoted as *B_reg_*, *N_reg_* and *D_reg_*, respectively, and the whole TMI is denoted as *G_reg_*. Figure 3 shows three TMIs from ESPL-LIVE HDR database [34] and the corresponding brightness segmentation images, where the parts of red, green and blue in images are *B_reg_*, *N_reg_* and *D_reg_*, respectively. Obviously, it can be found that different TMIs appeared to have detail loss with different degrees in the three brightness regions. Then, these segmented images were regarded as region masks for the following block extraction with DB-regions. Specifically, TMIs were divided into multiple non-overlapping blocks with the same size, and these blocks were categorized as three subsets via the obtained region masks. Finally, each image in the TMI training dataset contained four kinds of blocks, that is, $T=\{T^{B_{reg}},T^{N_{reg}},T^{D_{reg}},T^{G_{reg}}\}$, where $T^{B_{reg}}$, $T^{N_{reg}}$, $T^{D_{reg}}$ and $T^{G_{reg}}$ are the blocks in *B_reg_*, *N_reg_*, *D_reg_* and *G_reg_*, respectively.

#### 2.1.3. Multi-Dictionary Learning

To conduct regional sparse representation, multi-dictionaries based on brightness segmentation were obtained first, which contained three regional dictionaries and one global dictionary. At present, several dictionary learning algorithms have been proposed, and the principle idea is to find out a set of representative atoms that can approach the training data optimally on the condition of specific sparse constraint. Generally, let $D^{r}\in R^{n\times m}$ denote an over-complete dictionary, where *m* is the number of atoms and every atom is an *n*-dimensional vector. Let $T={\left\{T_{i}\right\}}_{i=1}^{N}$ denote the predivided TMI blocks with the size of $\sqrt{n}\times \sqrt{n}$ from the collected multi-dictionary sets, where ***T****_i_* represents the *i*-th block of DB-regions or the global region, $T_{i}\in R^{n}$, and *N* is the total number of blocks. In short, the input source of dictionary learning is sampled from image block samples. In the proposed method, taking $T^{r}$ as the input, the multi-dictionaries $D^{r}$ are obtained by solving an optimization problem. Then, the optimization scheme can be defined as

(1) $$\langle D^{r},S^{r}\rangle =\arg\min{\left\|s_{i}^{r}\right\|}_{0},s.t.{\left\|T^{r}-D^{r}S^{r}\right\|}_{2}^{2}\leq t$$

where $S^{r}={\left\{s_{i}^{r}\right\}}_{i=1}^{N}$ are the sparse coefficient of $T^{r}$ acquired by $D^{r}$, $r\in \left\{B_{reg},N_{reg},D_{reg},G_{reg}\right\}$ denotes the category of region and *t* is the initial error threshold, which is set to 5 empirically. Here, the K-SVD algorithm [36] is selected to solve the optimization scheme in Equation (1) due to its fast solution and strong competitiveness.

**Figure 4:**
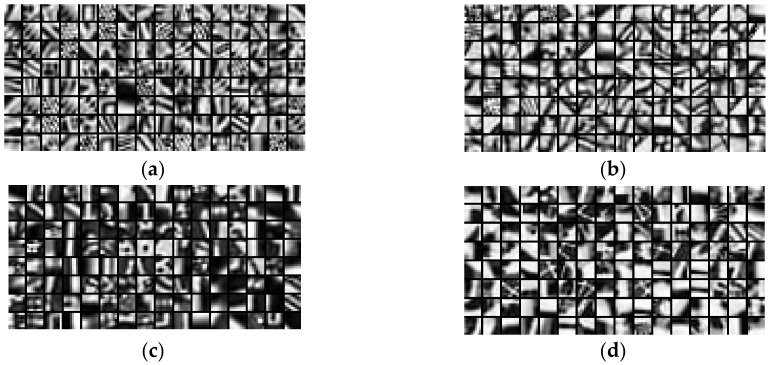
The learned multi-dictionaries. (**a**) The dictionary learned by normal exposure blocks; (**b**) the dictionary learned by bright blocks; (**c**) the dictionary learned by dark blocks and (**d**) the dictionary learned by all blocks.

To obtain the regional sparse responses, the multi-dictionaries about *B_reg_*, *N_reg_*, *D_reg_* and *G_reg_* were obtained as shown in Figure 4. It can be found that the dictionary trained by $T^{N_{reg}}$ contained more details than the global dictionary, while the dictionary of $T^{B_{reg}}$ had the minimal visual information, as well as the dictionary of $T^{D_{reg}}$. In conclusion, each atom in the multi-dictionaries captured visual information of DB-regions, which was in accordance with the perceptual characteristics of TMI. Remarkably, the learned multi-dictionaries were not required to be updated later and could be used directly as the target dictionaries for feature representation during the testing phase.

### 2.2. Regional Sparse Response Feature Extraction

#### 2.2.1. Sparse Atomic Activity in Each Region

Feature coding is an effective means to obtain a set of novel feature representations by transforming the original feature space into the target dictionary space, and the corresponding activity of each atom can be regarded as the final feature code. Here, we made a series of analyses to quantify the detail distortion of DB-regions. Firstly, we divided the distorted TMI into multiple non-overlapping blocks with the same ways in the previous dictionary learning stage, and categorized them as four subsets ${\hat{T}}^{r}$ via the calculated region masks. Each kind of block can be sparsely represented with the multi-dictionaries $D^{r}$ to obtain the corresponding feature codes, and the above process is expressed as

(2) $${\hat{X}}^{r}=\arg\min{\left\|{\hat{x}}_{i}^{r}\right\|}_{0},s.t.{\left\|{\hat{T}}^{r}-D^{r}{\hat{X}}^{r}\right\|}_{2}^{2}\leq t$$

where ${\hat{X}}^{r}={\left\{{\hat{x}}_{i}^{r}\right\}}_{i=1}^{N}$ is the estimated sparse coefficient based on block representation. $r\in \left\{B_{reg},N_{reg},D_{reg},G_{reg}\right\}$ denotes the category of region, and the orthogonal matching pursuit (OMP) algorithm is used to solve the optimization problem in Equation (2).

Actually, the obtained sparse coefficients can characterize the activity type of atoms in DB-regions, so exploring the potential statistical rules of sparse coefficients is considered as a meaningful way for feature representation. Since the sparse coefficients are made up of many values, for brevity, let *SC_coeff_* denote the all sparse coefficients, *SC_coeff-l_* denote the sparse coefficients of less than zero and *SC_coeff-g_* denote the sparse coefficients of greater than zero. Moreover, *SC_coeff-l_* and *SC_coeff-g_* are extracted by setting the other type of coefficients (i.e., *SC_coeff-g_* and *SC_coeff-l_*) to zero for analyzing their contributions on sparse representation, respectively. Then, image reconstruction was conducted by only using one type of coefficients to observe the restored TMIs and the corresponding histogram distribution.

**Figure 5:**
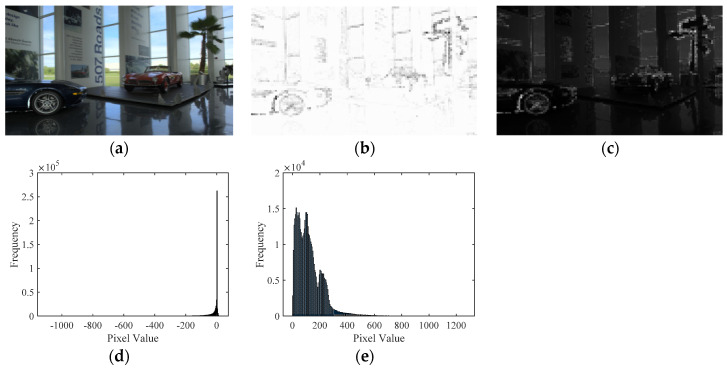
Original TMI and its reconstructed versions with different sparse coefficients. (**a**) Original TMI; (**b**) the reconstructed image with sparse coefficients greater than 0; (**c**) the reconstructed image with sparse coefficients less than 0; (**d**) corresponding histogram of the image in (**b**,**e**) corresponding histogram of the image in (**c**).

**Figure 6:**
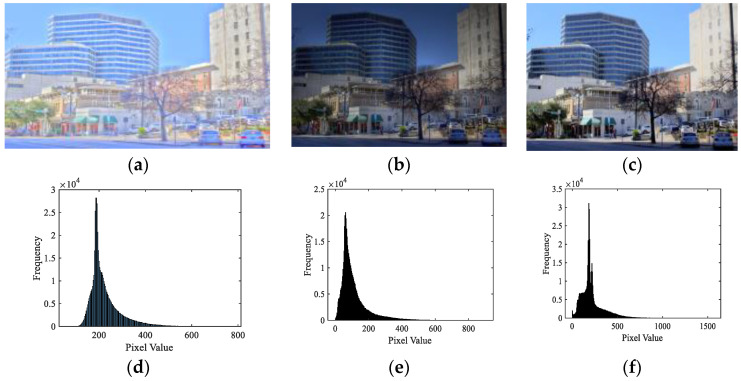
Three TMIs from the ESPL-LIVE HDR database [29] and the corresponding histograms of reconstructed images with *SC_coeff-l_*. (**a**) TMI generated by DurandTMO (mean opinion score (MOS) = 27.10); (**b**) TMI generated by FattalTMO (MOS = 47.99); (**c**) TMI generated by ReinhardTMO (MOS = 60.30) and (**d**–**f**) corresponding histograms of reconstructed images in the first row. Note that a higher MOS indicates a better quality in ESPL-LIVE HDR.

Figure 5 depicts an example of the reconstructed results with different sparse coefficients obtained by the global dictionary. From Figure 5, it can be found that the image reconstructed by *SC_coeff-l_* contained more information of the original TMI compared with the image reconstructed by *SC_coeff-g_*, which indicates that the atomic energy was mostly concentrated in *SC_coeff-l_*. To further illustrate the significant role of *SC_coeff-l_* for identifying the detail loss of DB-regions in TMI, we selected three TMIs generated by different TMOs and reconstructed them with the corresponding *SC_coeff-l_*. The reconstructed results and histogram statistics are shown in Figure 6. Obviously, the better the quality of TMI (i.e., the higher mean opinion score (MOS)), the wider the histogram pixel range of reconstructed image, which was consistent with the fact that high-quality TMI could maintain the detail information of its original HDR image as much as possible. Since *SC_coeff-l_* could reconstruct the image well, the distortion information will also be mainly reflected in *SC_coeff-l_*, and some redundancy could be eliminated by aggregating the features with *SC_coeff-l_*.

According to the above analysis for the sparse atom, visual information could be simply quantified by the activity statistics of *SC_coeff-l_*, which is expressed as:

(3) $${\hat{X}}^{r^{\prime }}=\left[{\hat{x}}_{1}^{r^{\prime }},{\hat{x}}_{2}^{r^{\prime }},\ldots ,{\hat{x}}_{N}^{r^{\prime }}\right]\in R^{m\times N}$$

(4) $$F_{1}^{r^{\prime }}=\overset{N}{\underset{T=1}{\sum }}g\left({\hat{x}}_{T}^{r^{\prime }}\right)$$

where ${\hat{x}}_{}^{r^{\prime }}$ is the obtained sparse feature vector for each image block, $r^{\prime }\in \left\{B_{reg},N_{reg},D_{reg}\right\}$ denotes the category of region, $g\left(.\right)$ is the function for counting the frequency of *SC_coeff-l_*. If ${\hat{x}}_{{}_{T}}^{r^{\prime }}$ is less than zero, $g\left({\hat{x}}_{{}_{T}}^{r^{\prime }}\right)$ is 1, otherwise, $g\left({\hat{x}}_{{}_{T}}^{r^{\prime }}\right)$ is 0. $F_{1}^{r^{\prime }}$ is the calculated activity statistical features, and the smaller value of $F_{1}^{r^{\prime }}$ indicates the lower activity of the corresponding region. For some TMIs without any dark or bright blocks, the corresponding sparse coefficients in the bright or dark region were zero. When they were stimulated by the visual primitive, these regions could not generate efficient responses. Therefore, gathering the features $F_{1}^{r^{\prime }}$ of DB-regions is an effective means to solve the difficulty caused by no response.

To aggregate the sparse features extracted from three brightness regions, i.e., $F_{1}^{B_{reg}}$, $F_{1}^{N_{reg}}$ and $F_{1}^{D_{reg}}$, we also designed a novel regional feature fusion strategy based on entropy weighting, which was inspired by the evidence that entropy could reflect the visual information contained in images to some extent. First, each block in the DB-regions was rearranged into a vector with the length of *n* and the corresponding blocks were aggregated to obtain the matrices $M^{r^{\prime }}$. Then, the entropy weight $w^{r^{\prime }}$ for each region was computed as

(5) $$w^{r^{\prime }}=\frac{E^{r^{\prime }}}{\sum E^{r^{\prime }}}$$

where $E^{r^{\prime }}$ is the obtained entropy of DB-regions by applying the entropy calculation operation to $M^{r^{\prime }}$.

Finally, the optimized sparse atomic activity statistics features ***F***_1_ could be calculated as:

(6) $$F_{1}=\sum w^{r^{\prime }}F_{1}^{r^{\prime }}$$

where the dimension of ***F***_1_, namely *m*, was set to 128, and we will give the specific explanations in Section 3.

#### 2.2.2. Global Reconstruction Residual Statistics

In general, HVS first focuses on the global perception of an image unconsciously, and gradually turns to some specific local regions [26]. In terms of global perception, high-quality TMI should contain rich detail components and high naturalness, which are especially reflected in high frequency information. Considering that residual information of images play a significant role in distortion recognition, we performed the statistical analysis on global residual image for perceiving global detail loss of TMI, and global residual image *I*_g_ can be simply obtained by calculating the difference between the reconstructed TMI with pretrained global dictionary $D^{G_{reg}}$ and original TMI.

Furthermore, the mean subtracted contrast normalized (MSCN) coefficients of the image appears to have a certain statistical rule, that is, when an image is impaired with single or multiple distortion, the relevant natural statistical distribution of the MSCN coefficient will be destroyed. Therefore, the MSCN operation was conducted first on the global residual image *I*_g_ to quantize the distortion, which is expressed as

(7) $$\hat{I}\left(i,j\right)=\frac{I_{g}\left(i,j\right)-\mu \left(i,j\right)}{\sigma \left(i,j\right)+1}$$

where $\hat{I}\left(i,j\right)$ is the MSCN value of *I*_g_ at the position of $\left(i,j\right)$, $\mu \left(i,j\right)$ and $\sigma \left(i,j\right)$ are the local mean and standard deviation of *I*_g_, respectively.

**Figure 7:**
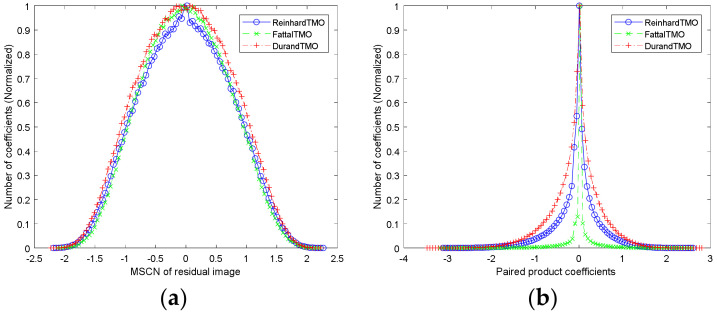
The histograms of the mean subtracted contrast normalized (MSCN) coefficients and paired product coefficients of residual image under three TMOs. (**a**) Histogram of MSCN coefficients and (**b**) histograms of paired products of MSCN coefficients.

Figure 7a depicts three histograms of MSCN coefficients under different TMOs (original images are shown in Figure 6a–c, respectively). It can be found that the histograms of MSCN coefficients of residual images present a statistical rule similar to Gaussian distribution, and have obvious distinguishing ability for different TMOs. Therefore, generalized Gaussian distribution (GGD) was utilized to match these MSCN coefficients effectively in this work, and the density function of GGD with zero mean is given by

(8) $$f\left(x;\alpha ,\sigma^{2}\right)=\frac{\alpha }{2\beta \Gamma \left(1/\alpha \right)}\exp[-\left(\left|x\right|/\beta {)}^{\alpha }\right]$$

where $\beta =\sigma \sqrt{\Gamma \left(\alpha^{-1}\right)/\Gamma \left(\alpha^{-3}\right)}$, $\Gamma \left(.\right)$ is the standard Gamma function. α and $\sigma^{2}$ controls the shape and variance of Gaussian distribution, respectively. The two control parameters $\left(\alpha ,\sigma^{2}\right)$ constitute the first set of compensation features for detecting the global detail loss of TMIs.

In addition, we also explored the statistical rules among the neighboring pixels of residual image, and the relevant pairwise products of neighboring MSCN coefficients along four directions were calculated as

(9) $$H\left(i,j\right)=\hat{I}\left(i,j\right)\hat{I}\left(i,j+1\right)$$

(10) $$V\left(i,j\right)=\hat{I}\left(i,j\right)\hat{I}\left(i+1,j\right)$$

(11) $$D_{1}\left(i,j\right)=\hat{I}\left(i,j\right)\hat{I}\left(i+1,j+1\right)$$

(12) $$D_{2}\left(i,j\right)=\hat{I}\left(i,j\right)\hat{I}\left(i+1,j-1\right)$$

where $H\left(i,j\right)$, $V\left(i,j\right)$, $D_{1}\left(i,j\right)$ and $D_{2}\left(i,j\right)$ characterize the statistical relationships along the horizontal, vertical, main diagonal and subdiagonal directions, respectively. Figure 7b is three histograms of paired products of MSCN coefficients under different TMOs, which also shows strong distortion identification of adjacent coefficients. To fit the above presented regular structure accurately, asymmetric generalized Gaussian distribution (AGGD) is applied in each pairwise product of coefficients, which is defined as

(13) $$f\left(x;v,\sigma_{l}^{2},\sigma_{r}^{2}\right)=\left\{\begin{array}{l} \frac{v}{\left(\beta_{l}+\beta_{r}\right)\Gamma \left(v^{-1}\right)}\exp\left(-{\left(\frac{-x}{\beta_{l}}\right)}^{v}\right),x<0\\ \frac{v}{\left(\beta_{l}+\beta_{r}\right)\Gamma \left(v^{-1}\right)}\exp\left(-{\left(\frac{-x}{\beta_{r}}\right)}^{v}\right),x\geq 0 \end{array}\right.$$

(14) $$\eta =\left(\beta_{r}-\beta_{l}\right)\frac{\Gamma \left(2/v\right)}{\Gamma \left(1/v\right)}$$

where $\beta_{l}=\sigma_{l}\sqrt{\Gamma \left(v^{-1}\right)/\Gamma \left(v^{-3}\right)}$ and $\beta_{r}=\sigma_{r}\sqrt{\Gamma \left(v^{-1}\right)/\Gamma \left(v^{-3}\right)}$. v controls the shape of the distribution, $\sigma_{l}^{2}$ and $\sigma_{r}^{2}$ are two scale parameters. The control parameters $\left(\eta ,v,\sigma_{l}^{2},\sigma_{r}^{2}\right)$ of each paired product yield the second set of compensation features, and are combined with $\left(\alpha ,\sigma^{2}\right)$ to form the global reconstruction residual statistical features, denoted as $F_{2}$, whose dimension is 36.

In conclusion, the final regional sparse response features consist of two types of feature sets, i.e., sparse atomic activity and global reconstruction residual statistics, which describe the regional and global visual information in the sparse domain, respectively.

### 2.3. Aesthetic Feature Extraction

Although the trend of the presented regional sparse response features is roughly in accordance with the subjective perception of TMIs caused by detail loss, the other perceptual factor in TMI (i.e., color) cannot be ignored due to the color unnaturalness of scenes, as depicted in the first row of Figure 6. It can be clearly observed that different visual effects would be produced by different TMOs under the same HDR image. For example, the MOS of TMI generated by DurandTMO with relatively bright but unnatural color was lowest, while the TMI obtained by ReinhardTMO reflected higher contrast than others and had the highest MOS value. Therefore, for the whole TMI, some perceptual features, such as global contrast, color fidelity, color temperature and darkness, were also extracted in this subsection, which are jointly called as aesthetics.

#### 2.3.1. Global Contrast

Contrast tends to reflect the relationship among pixels, which cannot be clearly expressed by the sparse coefficient in the sparse domain and HVS will pay more attention to the overall contrast of the image than the absolute brightness. Therefore, Michelson contrast *C*_m_ and root mean square *C*_rms_ were selected to characterize the overall contrast relating to the color naturalness of TMI, and the above features were extracted in the HSI color space, which is expressed as

(15) $$C_{m}=\frac{I_{\max}-I_{\min}}{I_{\max}+I_{\min}}$$

(16) $$C_{\mathrm{rms}}=\sqrt{\frac{1}{WH}\overset{H-1}{\underset{i=0}{\sum }}\overset{W-1}{\underset{j=0}{\sum }}{\left(I_{ij}-\bar{I}\right)}^{2}}$$

where $I_{\max}$ and $I_{\min}$ were the largest and smallest pixel values of the image, respectively. *W* and *H* are the width and height of TMI, respectively. $\bar{I}$ is the average value of pixels. The two parameters $\left(C_{m},C_{\mathrm{rms}}\right)$ form the global contrast feature set and denoted as 2-dimensional ***F***_3_.

#### 2.3.2. Color Fidelity

When contrast is guaranteed, color fidelity is also considered as an important feature to capture the color saturation of TMIs, which can be simply calculated by image color invariance descriptors. Since LMS space can simulate the response of cones in the retina and the three types of LMS cones can correspond to two opposing colors, which is called antagonism [37], we transformed the image data into the logarithmic domain so that three major orthogonal non-correlated spines (denoted as l, ς and τ) were computed by

(17) l=L^+M^+S^3,ς=L^+M^−2S^6,τ=L^−M^2

where $\hat{L}$, $\hat{M}$ and $\hat{S}$ are the L, M and S channels after logarithm operation, deaveraging and normalization, respectively. Surprisingly, similar statistic rules are presented in the histograms of l, ς and τ coefficients, which can also be fit by GGD. Therefore, six parameters (the shape and variance parameters of the three channels) form the color fidelity feature set and denoted as 6-dimensional ***F***_4_.

#### 2.3.3. Color Temperature

Color temperature reflects the spectral composition of the light source and has been applied successfully in many fields, such as photography, video recording and publishing. Generally, the level of color temperature will directly affect the brightness and contrast of images, which is closely related to the color perception of the light source. Therefore, color temperature [38] is used to detect the color unnaturalness of TMIs in this study, and can be defined as

(18) $$C_{\mathrm{CCT}}=\left(449p^{3}+3525p^{2}+6823.3p+5520.33\right)$$

where

(19) $$p=\frac{a-0.3320}{0.1858-b},a=\frac{X}{X+Y+Z},b=\frac{Y}{X+Y+Z}$$

Among them, *X*, *Y* and *Z* represent the three-channel values of XYZ color space converted from RGB color space. Then, 5-bin histogram statistics were performed on the obtained color temperature map, and five frequency values were taken as the final color temperature feature set, denoted as ***F***_5_, whose dimension was 5.

#### 2.3.4. Darkness

Darkness depicts the proportion of pixels with low brightness values in the image, and has a great impact on color unnaturalness. If the whole image looks dim, its subjective quality perceived by HVS is more terrible than ones with perfect brightness. Inspired by the three-point method in camera science, a TMI was first evenly divided into three blocks from top to bottom, and the mean pixel value of TMI was calculated. Then, the proportions of three blocks and the whole image whose brightness was less than the mean pixel value were computed and these four values were used as the final darkness feature set, denoted as 4-dimensional ***F***_6_.

### 2.4. Quality Regression

In brief, a total of 181-dimensional quality-aware features were extracted from a TMI via regional sparse response and aesthetics analysis, denoted as ***F*** = {***F***_1_, ***F***_2_, ***F***_3_, ***F***_4_, ***F***_5_, ***F***_6_}, where the former two were regional sparse response features and the other four are aesthetic features. After feature extraction, the feature space was mapped to predict the quality Q of TMI by quality regression, which is expressed as

(20) $$Q=\psi \left(F\right)$$

where $\psi \left(.\right)$ is the mapping function achieved by machine learning. Due to the strong prediction accuracy of random forest (RF), RF was used to obtain the mapping function in this study.

## 3. Experiment Results and Discussion

To verify the performance of the proposed RSRA-BTMI method, the ESPL-LIVE HDR [34] database was used to make comparisons between the proposed method and existing state-of-the-art BIQA methods. The database was generated by three different types of HDR image processing algorithms, including TMO, multi-exposure fusion and post-processing. The images processed by TMOs and their corresponding subjective scores were utilized in the experiment. The basic situation of TMIs in the ESPL-LIVE HDR database is shown in Table 1. It contained a total of 747 TMIs degraded by TMOs.

In order to validate the accuracy of the method, 80% of the image samples in the database were selected as the training set to train a TM-IQA model, which was used to predict the quality of the remaining 20% image samples. The scenarios of the training set and testing set were independent of each other. Then, to evaluate whether the method is statistically consistent with visual perception, it is necessary to compare the predicted scores with subjective ratings. According to the objective IQA standard proposed by the Video Quality Expert Group (VQEG), Pearson linear correlation coefficient (PLCC), Spearman rank-order correlation coefficient (SROCC) and root mean squared error (RMSE) were employed to validate the consistence. With experience, a method correlates well with subjective scores if PLCC and SROCC are close to 1 and RMSE is close to 0. In addition, to get the reliable results of the proposed RSRA-BTMI method, the above procedure was repeated 1000 times using randomly divided training and testing sets. Finally, we reported the median value of performance index obtained from the 1000 random trails as the final performance index.

### 3.1. Parameter Setting and Feature Analysis of the Proposed RSRA-BTMI Method

As can be found from the feature extraction in Section 2, the size of some parameters needs to be set. Actually, the size of the presegmented block of TMI for dictionary learning will affect what the block contains. Specially, the larger the block, the greater the probability that the block contains different luminance content, and the operation of block based regional subset partition is more difficult. This will affect multi-dictionary learning and accurate extraction of sparse feature vector. However, the smaller of blocks will cause the higher complexity and lower efficiency of the proposed method. Therefore, the block size is set to a moderate value 8 × 8, and the dictionary size *m* is set to 128. *m* also determines the size of the final feature vector, so the feature size extracted from each region in the sparse domain is 128.

As described in Section 2, there were several types of features extracted in this work. Sparse atomic activity based on regional entropy weighting ***F***_1_ and auxiliary statistics based on global reconstruction residual ***F***_2_ represent regional sparse response features in the sparse domain. Contrast ***F***_3_, color fidelity ***F***_4_, color temperature ***F***_5_ and darkness ***F***_6_ constitute the aesthetic features. Actually, most of the components in the sparse eigenvector were zero, and the non-zero component justified that the sample TMI had a corresponding response in the pretrained dictionary prototype. From a biological point of view, there were a series of visual neurons in the mammalian visual system. Visual neurons can sparsely encode the stimulus, that is, when a specific external stimulus is received, the information carried by the stimulus can be correctly perceived, as long as a small number of corresponding neurons accept the stimulus. Therefore, the sparse representation coefficients based on multi-dictionaries characterize the neuron state under a particular stimulus. The non-zero positions indicate that the neuron receives the stimulus, and the zero portions indicate that the neuron is not stimulated. Therefore, the sparse decomposition process of images is a sparse response of a neuron to a specific stimulus. A TMI to be assessed is transformed into sparse coefficients, and the sparse characteristics of each coefficient contain the essential features of the TMI. The feature extraction from the sparse domain will be more visually perceptible than the original image pixels. The more *SC_coeff-l_* represents that the more stimuli are received. To percept the global distortion, the global reconstruction residual statistics feature ***F***_2_ was extracted to assist ***F***_1_. The aesthetic features ***F***_3_, ***F***_4_, ***F***_5_ and ***F***_6_ were also considered because color distortion is not negligible in TMIs.

To analyze the feature contribution, the performances of each type of features were separately evaluated on the ESPL-LIVE HDR database. In addition, the combination contribution of ***F***_1_ and ***F***_2_ in the sparse domain was also given to confirm the validity of the proposed features, as well as the combination of aesthetic features ***F***_3_, ***F***_4_, ***F***_5_ and ***F***_6_. PLCC, SROCC and RMSE were used as the performance criteria. These results are shown in Table 2. We could observe that the separate feature shows good performance alone, and a better performance could be achieved when the features were incorporated together. This makes us believe that the proposed features are complementary with each other.

In the previous analyses in Section 2, it can be known that *SC_coeff-g_* had less effect on sparse reconstruction, but whether it had the ability to distinguish a high or poor-quality of the image or not remains to be validated. By the same proposed process of sparse atomic activity feature extraction in Section 2, sparse atomic activity statistics of different portions, such as *SC_coeff-g_* and the combination of *SC_coeff-l_* and *SC_coeff-g_,* were used to measure the performance for quality assessment. In Table 3, *SC_coeff-lg_* is represented for the combination of *SC_coeff-l_* and *SC_coeff-g_*.

Table 3 lists three types of features about the activity of *SC_coeff-g_*, *SC_coeff-lg_* and *SC_coeff-l_*. It can be found that *SC_coeff-g_* and *SC_coeff-lg_* also exhibited good quality discrimination performance, and even exceeded the performance of the methods such as BTMQI, which will be shown later. According to the comparison, the portion of *SC_coeff-l_* was selected as the final fusion feature in the sparse domain.

In addition, to verify the advantage of multi-dictionaries in the proposed RSRA-BTMI method, Table 4 lists the experimental analysis of single dictionary and multi-dictionaries. In Table 4, the performance obtained by combining multi-dictionaries with aesthetic characteristics was better than that obtained by combining single dictionary with aesthetic characteristics, here, they were denoted as ‘M + A’ and ‘S + A’, respectively. It is mainly attributed that those multi-dictionaries take more account of the different characteristics of HDR images after the TM process in DB-regions. Together with aesthetics, it can better perceive the detail loss in the DB-regions and color unnaturalness.

**Figure 8:**
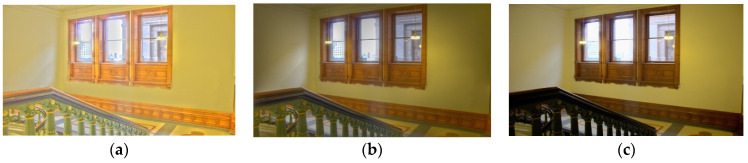

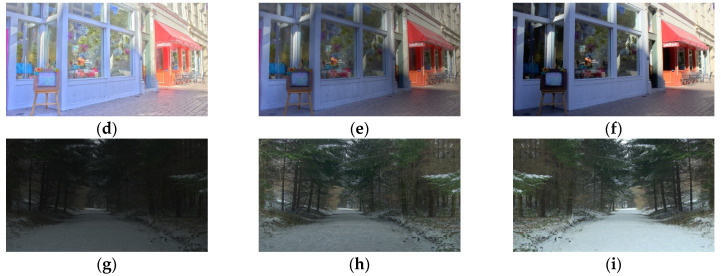
The predicted quality (i.e., Q) of different TMIs with their corresponding MOS values. (**a**) MOS = 35.06; Q = 35.1305; (**b**) MOS = 44.61; Q = 48.3781; (**c**) MOS = 49.23; Q = 51.1368; (**d**) MOS = 35.14; Q = 34.1305; (**e**) MOS = 44.51; Q = 48.7145; (**f**) MOS = 52.41; Q = 53.9768; (**g**) MOS = 40.64; Q = 37.7629; (**h**) MOS = 54.86; Q = 51.2037 and (**i**) MOS = 59.71; Q = 56.8727.

To clearly show a high correlation of aesthetic features with subjective scores, we trained a quality prediction model by aesthetic features. According to the trained quality prediction model, we used the aesthetic features of different distorted TMIs to predict the quality, the results are shown in the following Figure 8. It can be found that the more natural TMI is, the higher the predicted quality value (i.e., Q) will be, and also a companion with a higher MOS value.

### 3.2. Influence of Training Set Sizes

In order to study the influence of different training sets on quality prediction results, PLCC and SROCC values obtained via different training sets were also analyzed, as shown in Table 5. The training set size was set as 10–90%, and we could draw the following conclusions via the results in the Table 5: (1) with the increasing of the training set, PLCC and SROCC values also increased gradually, which is consistent with the conclusion of the existing learning-based BIQA method and (2) when the training set was less than 20%, the performance dropped significantly, but it also had better performance than other existing methods, such as BTMQI shown in the Table 6.

### 3.3. Feature Selection

**Figure 9:**
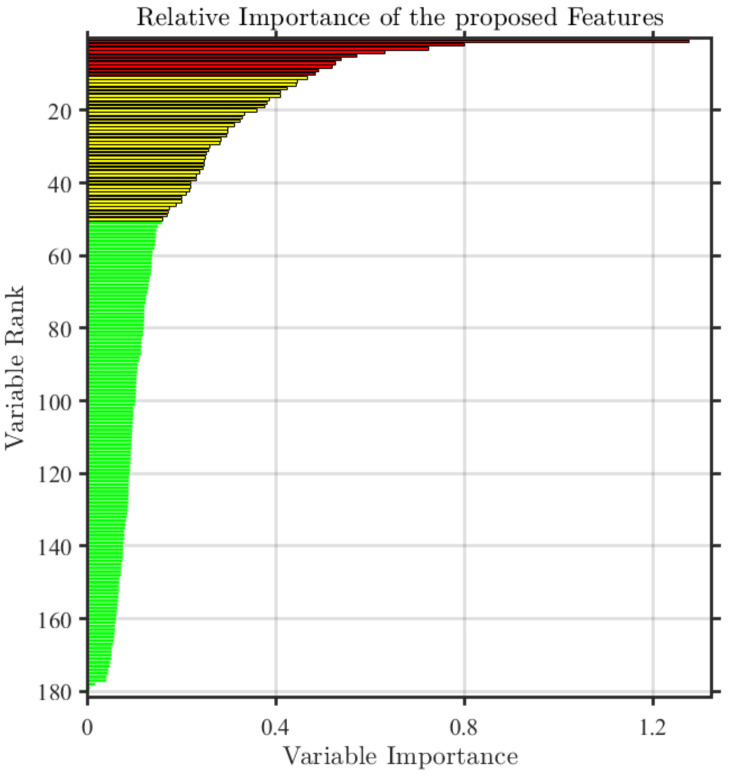
Importance ranking of different features.

**Figure 10:**
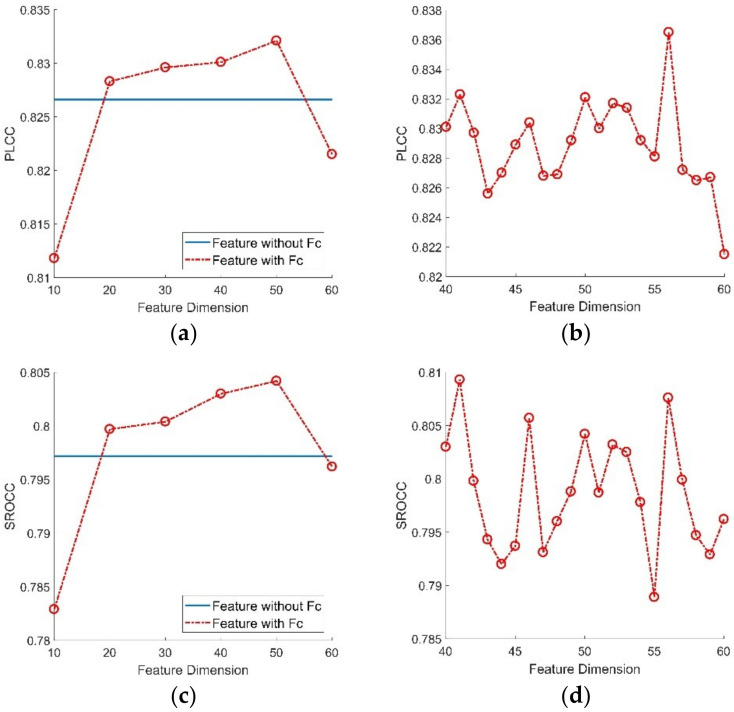
Performance of Pearson linear correlation coefficient (PLCC) and Spearman rank-order correlation coefficient (SROCC) values with different feature dimensions. (**a**) PLCC value of 10–60 dimensional features; (**b**) PLCC value of 40–60 dimensional features; (**c**) SROCC value of 10–60 dimensional features and (**d**) SROCC value of 40–60 dimensional features.

Since the total 181-dimensional features may cause an overfitting situation, we made an experiment to eliminate redundancy from the total features. RF has an ability to detect the importance of features, so it can well guide the feature selection work. Specifically, we utilized RF to predict the importance of features extracted in the ESPL-LIVE HDR database as shown in Figure 9 [23]. It can be found that different features had different importance. To determine the best feature dimension, we utilized different dimension of features to build quality prediction model and evaluate the corresponding performance. As shown in Figure 10, it can be found that the performance of PLCC and SROCC was best when the dimension of feature was 56. For brevity, the feature set after importance selection is expressed by ‘***F***_c_’ in the following description.

### 3.4. Overall Performance Comparison

In order to prove the effectiveness of the proposed RSRA-BTMI method, it was compared with the existing advanced BIQA methods. Since the ESPL-LIVE HDR database did not provide the original HDR reference image, the FR-IQA methods designed for TMIs could not be utilized on the database directly. The proposed RSRA-BTMI method was not compared with the existing FR-IQA methods. Table 5 shows the performance comparisons between the proposed RSRA-BTMI method and two types of existing IQA methods. The first type is the 2D-BIQA methods specialized for ordinary LDR images based on natural scene statistical features, including C-DIIVINE [14], DIIVINE [13], BLIINDS-II [15], BRISQUE [16] and OG [39]. The other type is specifically designed for TM-BIQA, including BTMQI [19], HIGRADE [21], Yue’s [22], BTMIQA [20], BLIQUE-TMI [23] and Chi’s [25].

From Table 6, it can be found that the performance of the TM-BIQA methods was far superior to the 2D-BIQA methods for TMIs’ quality assessment because the TMIs’ distortion types were different from those of ordinary LDR images. In general, the distortion of LDR images included some common distortions, such as encoding distortion, and Gaussian noise. However for a TMI, its distortion mainly reflected in the color unnaturalness and the detail loss especially in its *B_reg_* and *D_reg_*. Therefore, it is unsuitable to directly use the 2D-BIQA methods to evaluate the TMIs’ quality. First of all, obviously, as the 2D-BIQA methods, C-DIIVINE, DIIVINE, BLIINDS-II, BRISQUE and OG only consider the corresponding distortions of ordinary LDR images, such as JPEG, JP2K compression, blur, white noise, etc., the quality prediction performances of these 2D-BIQA methods used on TMIs were usually poor, and their PLCC and SROCC values were very low, only about 0.530 and 0.523 at the best. Secondly, the PLCC values of the existing TM-BIQA methods were much higher than those of the 2D-BIQA methods, as well as the SROCC values. Among the TM-BIQA methods, BTMQI mainly considered the details and structure preservation degree of TMIs, but did not consider the color distortion carefully, which had a great impact on the TMIs’ quality. HIGRADE also spares more effects on the structure and naturalness, but neglects the color distortion. The BTMIQA method mainly uses the local entropy to perceive the detail loss of TMIs but it omits the information loss in the normal exposure region. The other methods also have room for improvement. The proposed method applies sparse perception with multi-dictionaries to extract main features of TMI’s DB-regions, which not only can reduce visual redundancy but also obtain the human visual perception response to different regions. Moreover, it is clear that the proposed RSRA-BTMI method had better performance than the other methods. It is mainly due to the truth that the proposed RSRA-BTMI method utilizes the compressed sensing. Combining the regional sparse response with aesthetics can obtain the detail loss especially in *B_reg_* and *D_reg_* of TMIs, as well as the color distortion. Therefore, the proposed RSRA-BTMI method outperformed the existing methods and was consistent with the subjective perception of the human vision. It is also attributed to the fact that the proposed RSRA-BTMI method simulated the distortion process of TM in the sparse domain.

Moreover, we also calculated the performance after feature importance selection based on the proposed RSRA-BTMI method. Clearly, the feature selection used further improved the performance of the proposed RSRA-BTMI method.

### 3.5. Discussion

Due to the particularity of TMIs in imaging and viewing properties, two kinds of perceptual factors ought to be considered in the TM-BIQA method, i.e., detail loss and color unnaturalness. In this paper, we proposed an RSRA-BTMI method by considering the impact of DB-regions and the global region of TMI on human subjective perception, whose performance on the ESPL-LIVE HDR database was better than other competing 2D-BIQA and TM-BIQA methods. From the perspective of semantic invariance in DB-regions of TMIs, multi-dictionaries were specially designed so that each brightness region could be sparsely represented to describe the regional visual information. Moreover, global reconstruction residual statistics were also conducted to identify the high frequency information loss and utilized as the compensation features in the sparse domain. For the color unnaturalness, several color related metrics, such as contrast, color fidelity, color temperature and darkness, were analyzed and discussed carefully. As an efficient metric, the proposed RSRA-BTMI method could not only serve as the quality monitor in the end-to-end TMI processing pipeline, but also promoted the development of some relevant technologies, such as tone mapping, image enhancement and denoising of TMI.

Although the proposed RSRA-BTMI method achieved excellent results in evaluating TMIs degraded with detail loss and color distortion, there were still limitations in some respects. First, several special distortions may appear in the actual imaging process, e.g., abnormal exposure, violent noise and indelible artifacts. Obviously, the introduction of an artifact or noise will greatly increase the high frequency information of the image, but it is not belonging to the component of positive detail information in images and usually causes terrible visual perception. Therefore, the presented global reconstruction residual statistics will produce the opposite result in this special case. Second, for the proposed method, there is a blocking operation on TMI before multi-dictionary learning for DB-regions. However, fixed size blocks may result in regions of different brightness within one TMI block, which is not conducive to multi-dictionary learning. Thus, a more reasonable and efficient way to improve the application scope of the proposed RSRA-BTMI method is worth being explored.

## 4. Conclusions

In this paper, a blind tone-mapped image quality assessment method based on regional sparse response and aesthetics (RSRA-BTMI) was proposed by designing novel local and global feature subsets. It is mainly inspired by the fact that the detail loss and color unnaturalness phenomena in tone-mapped images (TMIs) were perceived by human visual system from global to local patterns. In terms of local features, multi-dictionaries were first trained from different brightness regions (DB-regions) in TMIs. Then the sparse atoms activities for DB-regions were calculated to portray regional visual information of TMIs. Finally, a regional feature fusion strategy based entropy weighting was designed to aggregate the above local features. In terms of global features, the statistics of residual information obtained by sparse representation was utilized as a compensation feature in the sparse domain, and a set of aesthetic features, such as contrast, color fidelity, color temperature and darkness, were also extracted to characterize the color unnaturalness of TMIs. Experimental results on the ESPL-LIVE HDR database demonstrated the superiority of the proposed RSRA-BTMI method. In future work, we are about to expand the practicability of dictionary learning and sparse representation for further exploring the perceptual factors in TMIs.

## Figures and Tables

**Table 1:** TMIs details of the ESPL-LIVE HDR database.

Database	Tone-Mapping Operator	Number of Images	MOS
ESPL-LIVE HDR	WardHistAdjTMO	181	0–100
	DurandTMO	187	0–100
	FattalTMO	187	0–100
	ReinhardTMO	192	0–100

**Table 2:** Performance of different feature types in the proposed method.

Features	TMIs			
	PLCC	SROCC	SROCC-STD	RMSE
*F*_1_	0.7163	0.6770	0.0435	7.1565
*F*_2_	0.6542	0.6001	0.0494	7.7211
*F*_1_ + *F*_2_	0.7782	0.7365	0.0379	6.4265
*F*_3_	0.7399	0.6916	0.0380	6.8578
*F*_4_	0.6532	0.6111	0.0490	7.7180
*F*_5_	0.6980	0.6272	0.0496	7.3233
*F*_6_	0.7306	0.6272	0.0498	7.0143
*F*_3_ + *F*_4_ + *F*_5_ + *F*_6_	0.8011	0.7678	0.0319	6.1003
All	0.8266	0.7972	0.0312	5.7520

**Table 3:** Performance of different sparse atomic activity features.

Activity of Different Sparse Coefficients	TMIs			
	PLCC	SROCC	SROCC-STD	RMSE
*SC_coeff-g_*	0.7098	0.6706	0.0438	7.2417
*SC_coeff-lg_*	0.7086	0.6698	0.0435	7.2410
*SC_coeff-l_*	0.7163	0.6770	0.0435	7.1565

**Table 4:** Performance comparison of multi-dictionaries and single dictionary with aesthetics.

Features	TMIs			
	PLCC	SROCC	SROCC-STD	RMSE
S + A	0.8058	0.7699	0.0330	6.0572
M + A	0.8266	0.7972	0.0312	5.7520

**Table 5:** Performance with different train-test sizes.

Train-Test	ESPL-LIVE	
	PLCC	SROCC
10–90%	0.7379	0.7054
20–80%	0.7714	0.7416
30–70%	0.7873	0.7562
40–60%	0.7961	0.7659
50–50%	0.7998	0.7684
60–40%	0.8056	0.7757
70–30%	0.8124	0.7813
80–20%	0.8266	0.7972
90–10%	0.8301	0.8043

**Table 6:** Performance comparison on the ESPL-LIVE HDR database.

Type	Methods	PLCC	SROCC	RMSE
2D-BIQA	C-DIIVINE [14,21]	0.453	0.453	9.167
	DIIVINE [13,21]	0.530	0.523	8.805
	BLIINDS-II [15,21]	0.4421	0.4120	9.330
	BRISQUE [16,21]	0.3701	0.3402	9.535
	OG [39]	0.4993	0.4899	8.8637
TM-BIQA	BTMQI [19,20]	0.6914	0.6778	/
	HIGRADE [20,21]	0.7940	0.7600	/
	Yue’s [22]	0.7422	0.7356	6.713
	BTMIQA [20]	0.8234	0.7835	/
	BLIQUE-TMI [23]	0.7120	0.7040	/
	Chi’s [25]	0.8301	0.7887	5.7193
	Proposed (RSRA-BTMI)	0.8266	0.7972	5.7520
	Proposed with *F*_c_ (RSRA-BTMI)	0.8365	0.8076	5.5408
